# Dihydroxyacetone phosphate accumulation leads to podocyte pyroptosis in diabetic kidney disease

**DOI:** 10.1111/jcmm.18073

**Published:** 2023-12-08

**Authors:** Zongwei Zhang, Hongtu Hu, Qiang Luo, Keju Yang, Zhengping Zou, Ming Shi, Wei Liang

**Affiliations:** ^1^ Division of Nephrology Renmin Hospital of Wuhan University Wuhan China; ^2^ Nephrology and Urology Research Institute of Wuhan University Wuhan China; ^3^ Department of Nephrology The Central Hospital of Wuhan Wuhan China; ^4^ Qianjiang Hospital Affiliated to Renmin Hospital of Wuhan University Qianjiang China; ^5^ Qianjiang Clinical Medical College Health Science Center Yangtze University Qianjiang China

**Keywords:** ALDOB, DHAP, diabetic kidney disease, glycolysis, mTORC1, podocyte, pyroptosis

## Abstract

Diabetic kidney disease (DKD) can lead to accumulation of glucose upstream metabolites due to dysfunctional glycolysis. But the effects of accumulated glycolysis metabolites on podocytes in DKD remain unknown. The present study examined the effect of dihydroxyacetone phosphate (DHAP) on high glucose induced podocyte pyroptosis. By metabolomics, levels of DHAP, GAP, glucose‐6‐phosphate and fructose 1, 6‐bisphosphate were significantly increased in glomeruli of db/db mice. Furthermore, the expression of LDHA and PKM2 were decreased. mRNA sequencing showed upregulation of pyroptosis‐related genes (*Nlrp3*, *Casp1*, etc.). Targeted metabolomics demonstrated higher level of DHAP in HG‐treated podocytes. In vitro, ALDOB expression in HG‐treated podocytes was significantly increased. siALDOB‐transfected podocytes showed less DHAP level, mTORC1 activation, reactive oxygen species (ROS) production, and pyroptosis, while overexpression of ALDOB had opposite effects. Furthermore, GAP had no effect on mTORC1 activation, and mTORC1 inhibitor rapamycin alleviated ROS production and pyroptosis in HG‐stimulated podocytes. Our findings demonstrate that DHAP represents a critical metabolic product for pyroptosis in HG‐stimulated podocytes through regulation of mTORC1 pathway. In addition, the results provide evidence that podocyte injury in DKD may be treated by reducing DHAP.

## 1 INTRODUCTION

Diabetes mellitus is a chronic disease that affects millions of people worldwide. Among its various complications, diabetic kidney disease (DKD) stands out as the most prevalent and severe complication associated with diabetes, serving as a primary factor behind end‐stage renal disease.^1^, ^2^, ^3^ DKD is characterized by progressive albuminuria, which occurs following the destruction of the glomerular filtration barrier.^1^, ^2^, ^3^ As one of the key structural components of this barrier, podocytes play a crucial role in maintaining glomerular permselectivity.^4^, ^5^ In diabetes, podocytes are subjected to injury through various pathogenic cues, including hyperglycaemia,^6^ oxidative stress^7^ and inflammation.^8^

Pyroptosis is a novel form of lytic regulated podocyte death that occurs in DKD. This process involves the activation of caspase‐1 and caspase‐4/−5/−11, which was activated by the inflammasome and the upregulation of gasdermin D (GSDMD) expression.^9^, ^10^ In response to diabetic conditions, mitochondrial reactive oxygen species (ROS) generation has been shown to initiate the inflammasome activation of NLRP3.^10^ The mechanistic target of rapamycin complex 1 (mTORC1) kinase regulates cell growth by maintaining the balance between anabolic and catabolic processes. It was found that hyperactivated mTORC1 plays a role in the pathogenesis of podocyte damage in DKD.^11^ Additionally, mTORC1 is involved in pyroptosis through regulation of ROS production.^12^, ^13^ However, whether mTORC1 activation is directly responsible for podocyte pyroptosis requires further investigation.

Hyperglycaemia, the most common and significant symptom of diabetes, can lead to various pathophysiological changes in podocytes, including abnormal glucose metabolism. Studies have shown that alterations in glycolysis flux and nutrient metabolism intermediates play crucial roles in the progression of DKD.^14^, ^15^, ^16^ During glycolysis, fructose‐6‐phosphate is converted into fructose 1,6‐bisphosphate (FBP) by phosphofructokinase, which is then cleaved into two triose phosphates: dihydroxyacetone phosphate (DHAP) and glyceraldehyde 3‐phosphate (GAP) by aldolase (ALDO). Previous research has suggested that elevated levels of DHAP are associated with renal injury in diabetic mice,^17^ and more recently, it was demonstrated that DHAP acts as a key metabolic molecule that can activate the mTORC1 pathway.^18^ Therefore, targeting the regulation of DHAP metabolism may be a potential therapeutic strategy for preventing or treating podocyte damage in DKD.

In this study, we examined the potential correlation between DHAP accumulation and mTORC1 activation in podocytes under diabetic conditions, both in vivo and in vitro. Additionally, we explored the impact of DHAP accumulation resulting from abnormal glycolysis processes on podocyte pyroptosis.

## 2 METHODS

### 2.1 Reagents and antibodies

For Western blotting, immunofluorescence (IF) staining, and immunohistochemical (IHC) staining, we utilized the following antibodies: anti‐ALDOB (GTX101363, GeneTex), anti‐LDHA (3582, CST), and anti‐PKM2 (4053, CST). The primary antibodies for Western blotting against NLRP3 (YT5382) was from Immunoway (USA). Anti‐pro Caspase1 + p10 + p12 (ab179515) was from Abcam (Cambridge, MA), primary antibodies for Western blotting against IL‐1β (12703), Phospho‐S6 (Ser235/236) (4858), S6 (2317), Phospho‐mTOR (Ser2448) (5536) and mTOR (2983) were from Cell signalling Technology (Danvers, MA). The primary antibodies for Western blotting against GSDMD (20770‐1‐AP) and α‐tubulin (11224‐1‐AP) were from proteintech (Wuhan, China). Anti‐GAPDH (sc‐365,062) was purchased from Santa Cruz (Santa Cruz, CA). We obtained the DAPI and secondary antibodies from Antgene (Wuhan, China) for our experiments.

### 2.2 Animal studies

CAWENS animal company (Changzhou, China) provided us with male db/m and db/db mice (8–9 weeks old, weighing 20–40 g), which was kept in specific pathogen‐free conditions at the Center for Animal Experiments of Wuhan University. The mice had unrestricted access to food and water throughout the study. All experimental procedures were conducted in accordance with the guidelines of the National Health and Medical Research Council of China and approved by the Animal Ethics Review Board of Wuhan University (Certificate Numbers: 20200306). Metabolic cages were employed to collect 24‐h urine samples from the mice. Urine albumin‐to‐creatinine ratio (ACR), blood sugar levels and body weight were measured biweekly. The mice were euthanized, and their kidneys were perfused with physiological saline for subsequent biochemical and renal pathological analysis. A portion of the kidneys was fixed in glutaraldehyde for electron microscope examination. The remaining kidney samples were stored for further analysis.

### 2.3 Glomerular isolation, mRNA sequencing and data analysis

The renal cortices of db/m and db/db mice were minced and subjected to collagenase digestion. Glomeruli were isolated from tubules using a sequential sieving method, following a previously described protocol.^17^ The mRNA sequencing data has been deposited in the GEO database under the accession number GSE184836.^17^

### 2.4 Metabolomics

The glomerular samples were separated using an Agilent 1290 Infinity LC Ultra high‐performance liquid chromatography (UHPLC) HILIC column. The column temperature was maintained at 25°C, and the flow rate was set at 0.5 mL/min. An injection volume of 2 μL was used. The mobile phase consisted of two components: (A) a mixture of water, 25 mM ammonium acetate, and 25 mM ammonia water; and (B) acetonitrile. The gradient elution procedure was as follows: 0–0.5 min, 95% B; 0.5–7 min, linear decrease in B from 95% to 65%; 7–8 min, linear decrease in B from 65% to 40%; 8–9 min, B held at 40%; 9–9.1 min, linear increase in B from 40% to 95%; 9.1–12 min, B held at 95%. Throughout the analysis, the samples were maintained at 4°C in an automatic sampler. The separation of the samples was performed using an Agilent 1290 Infinity LC UHPLC system and analysed using a Triple TOF 6600 mass spectrometer (AB SCIEX). Both electrospray ionization (ESI) positive and negative ion modes were employed for detection. Data S1 provides information on all the energy metabolites.

The harvested podocyte samples were stored at −80°C in a refrigerator, and targeted metabolomics analysis was performed following previous methods.^19^ In brief, mass spectrometry analysis was carried out using a 5500 QTRAP mass spectrometer (AB SCIEX) in negative ion mode. Multiple reaction monitoring mode was employed to detect the specific ion pairs of interest. The ion pair information for all energy metabolites can be found in Data S2.

### 2.5 Histology and immunohistochemistry examination

Kidney samples were promptly fixed in 4% paraformaldehyde (pH 7.4) for a duration of 24 h. Subsequently, the tissues were embedded in paraffin, sectioned at 5 μm thickness, and subjected to periodic acid‐Schiff stain (PAS) for visualization. Microscopic examination was performed using an Olympus microscope (Tokyo, Japan). For immunohistochemistry (IHC) staining, the sections were incubated with specific primary antibodies (LDHA, PKM2). To determine the percentage of positive staining area, five independent visual fields were selected from each experimental group, and Image J analysis software was utilized.

### 2.6 Cell culture and transfection

Dr. Moin A. Saleem from the Academic Renal Unit, Southmead Hospital, Bristol, UK, generously provided conditionally immortalized human podocytes, which were cultured under standard conditions. The culture medium comprised RPMI 1640 (HyClone, USA) supplemented with 10% heat‐inactivated fetal bovine serum (FBS; Gibco, USA), 100 U/mL penicillin G, 100 μg/mL streptomycin (Invitrogen, USA), and 1× insulin‐transferrin‐selenium (ITS; Invitrogen, USA). The podocytes were maintained at 33°C, and to induce differentiation, they were cultured at 37°C for 10–14 days without ITS. The differentiated podocytes were used in all experiments. To stimulate differentiation, the differentiated podocytes were exposed to 30 mM high glucose (HG) for 24 h. For siRNA transfection, Aldob siRNA (Qiagen) was transfected into the podocytes using HiPerFect transfection (Qiagen) following the manufacturer's instructions. The podocytes were seeded in six‐well plates, and when they reached 70% confluence, they were transfected with serum‐free medium containing 100 nM Aldob siRNA for 6–8 h. Afterward, they were recovered in complete medium and stimulated with 30 mM HG as needed. To overexpress ALDOB, the Aldob plasmid (Miaoling, Wuhan, China) was transfected into the podocytes using X‐tremeGENE HP DNA Transfection Reagent (Roche) following the manufacturer's instructions. A density of 2 × 10^5^ cells was seeded in each well of a six‐well plate and transfected with complexes containing 2 μg of Aldob plasmid and 2 μL of the X‐tremeGENE transfection reagent. The cells were then incubated at 37°C under normal conditions for 24 h, recovered in complete medium, and stimulated with 30 mM HG when necessary. Different concentrations of GAP (HY‐113054, MCE, China) ranging from 0 to 150 μg/mL were added to the complete medium. Additionally, 100 mM rapamycin (HY‐10219, MCE, China) was added to the complete medium. Each experimental result was confirmed using three independent podocyte clones.

### 2.7 Western blotting

Following treatment, the podocytes or kidney tissue were homogenized in RIPA lysis buffer supplemented with PMSF and a protease inhibitor cocktail (Roche) for 30 min at 4°C. The homogenates were loaded onto 8–10% SDS‐PAGE gels and subsequently transferred onto PVDF membranes. The membranes were blocked with 5% milk for 1 h. After blocking, the membranes were incubated overnight at 4°C with primary antibodies (LDHA, ALDOB, PKM2, p‐mTOR, mTOR, p‐S6, S6, NLRP3, GSDMD, IL‐1β, Caspase1, α‐tubulin and GAPDH). The following day, the membranes were incubated with secondary antibodies. After three washes, the protein bands were visualized using an ECL Chemiluminescent kit (Servicebio, China). Finally, the bands were analysed using the ChemiDocTM MP Imaging system (Bio‐Rad, USA).

### 2.8 Immunofluorescence staining

Following the treatment, the cells were fixed using 4% paraformaldehyde and subsequently blocked with 5% BSA. They were then incubated overnight at 4°C with specific primary antibodies (ALDOB). The cells were further incubated with fluorescent secondary antibodies for 1 h. After washing, the nuclei of the samples were counterstained with DAPI. The fluorescence results were analysed using a confocal laser microscope (Olympus, Japan).

### 2.9 DHAP level measurement

DHAP level was measured by the standard protocol of the kit (MAK275, Sigma‐Aldrich). In this assay, TPI converts DHAP to GAP, and the coupling enzyme reaction that occurs yields a fluorescent product (LEX = 535 nm/ LEM = 587 nm), which is proportional to the amount of DHAP. The DHAP level was calculated according to the absorbance (LEX = 535 nm/ LEM = 587 nm) measured by the enzyme plate tester (Perkin Elmer, USA).

### 2.10 TUNEL staining

To assess pyroptosis in podocytes derived from kidney tissue, TUNEL staining was performed using the manufacturer's instructions (Roche Applied Science, Germany). In brief, the staining area was treated with fresh DAB colour solution, and the colour development time was carefully monitored under a microscope. Positive nuclei were visualized as a brownish‐yellow colour, and the sections were subsequently washed with running water to halt the colour development process.

### 2.11 Levels of IL‐18 and IL‐1β in cultured supernatants

The levels of IL‐18 and IL‐1β in cultured supernatants was determined using specific ELISA kits (cat. nos. BMS267‐2, Invitrogen and BMS224‐2, Lifescience) according to the manufacturer's protocol. The absorbance was measured at 450 nm using an enzyme‐labelled analyser (Perkin Elmer, USA).

### 2.12 Lactate dehydrogenase release measurement

Podocytes were seeded in 96‐well plates at a concentration of 1 × 10^4^/ml. Lactate dehydrogenase (LDH) activity in podocytes released into the medium was assessed using an LDH cytotoxicity assay kit (Roche Diagnostics) according to the manufacturer's protocol.

### 2.13 ROS production assessment

To assess ROS production in glomeruli and podocytes, dihydroethidium (DHE, Invitrogen) and 2',7'‐dichlorodihydrofluorescein diacetate (H2‐DCFDA, Beyotime, China) assays were conducted.

### 2.14 Statistical analyses

The statistical process was performed with SPSS 22.0 software. All values are expressed as the mean ± standard deviation (SD). Differences between all groups were determined by Student's *t*‐test or one‐way analysis of variance (anova). *p* < 0:05 was considered statistically significant.

## 3 RESULTS

### 3.1 Glycolysis dysfunction and accumulation of DHAP in glomeruli of diabetic mice

**FIGURE 1:**
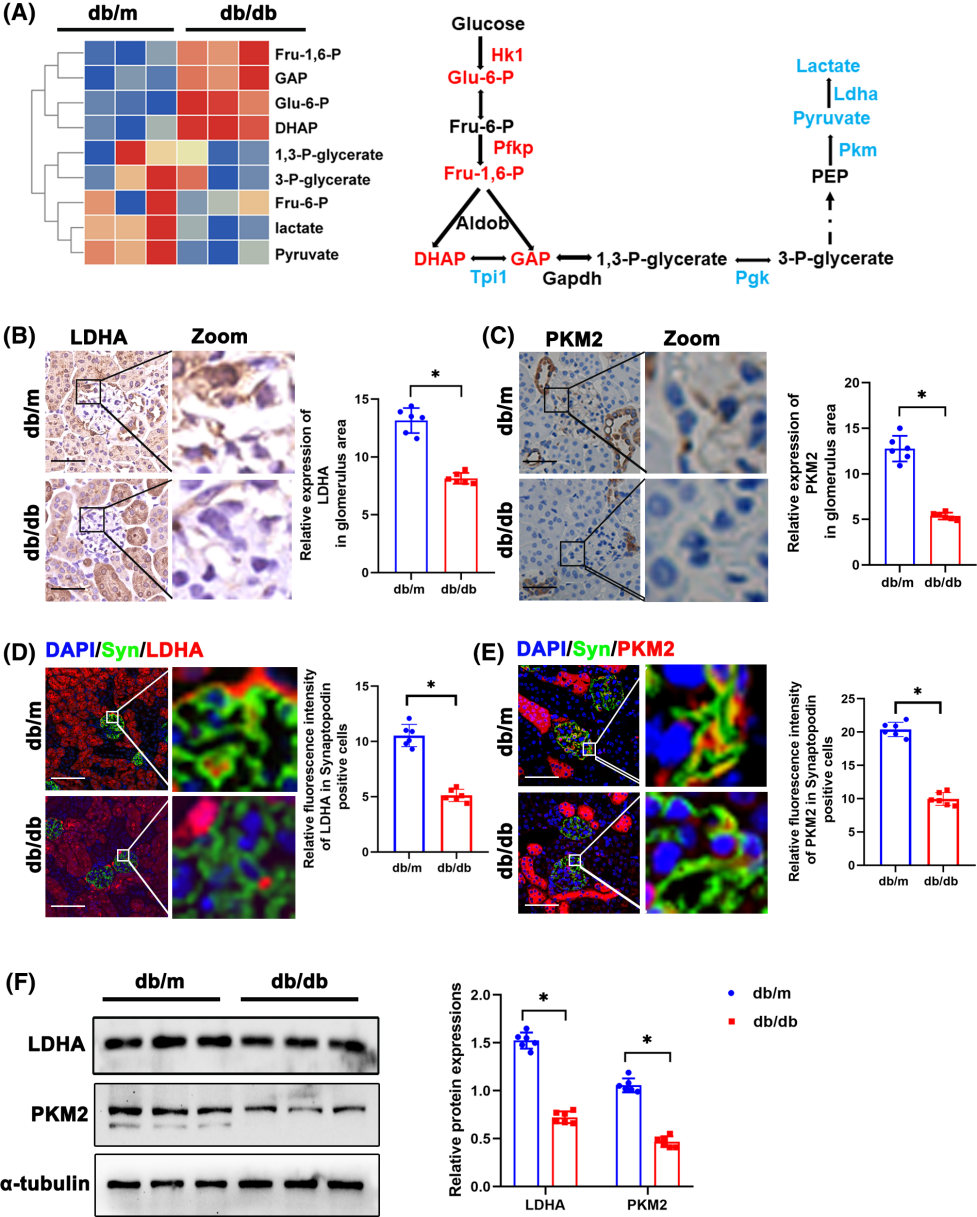
Glycolysis dysfunction and accumulation of DHAP in glomeruli of diabetic mice. (A)Metabolomics of glomeruli of db/m and db/db mice. Altered metabolites in glycolysis were showed in heat map. The schematic flow illustrates the representative genes and metabolites in glycolysis cascade. Letters in red represent upregulated genes and ones in blue represent downregulated metabolites from glomeruli of db/db mice in comparison to db/m mice. (B) Representative immunohistochemistry staining of glomerular LDHA in db/m and db/db mice. (C) Representative immunohistochemistry staining of glomerular PKM2 in db/m and db/db mice. (D) Representative immunofluorescent staining of LDHA in podocyte of glomeruli from db/m and db/db mice. Synaptopodin was used as podocyte markers. (E) Representative immunofluorescent staining of PKM2 in podocyte of glomeruli from db/m and db/db mice. Synaptopodin was used as podocyte markers. (F) Western blotting analysis of the expression of LDHA and PKM2 in glomeruli of db/m and db/db mice. α‐tubulin was used as loading control. Scale bars: 20 μm. *n* = 6. *p < 0.05. 1,3‐P‐glycerate, 1,3‐bisphosphoglycerate; 3‐P‐glycerate, 3‐phosphoglycerate; Aldob, aldolase B type; DHAP, dihydroxyacetone phosphate; Eno1, enolase 1; Fru 6‐P, D‐fructose 6‐phosphate; Fru‐1,6‐P, fructose 1, 6‐bisphosphate; GAP, Glyceraldehyde 3 phosphate; Gapdh, glyceraldehyde 3‐phosphate dehydrogenase; Glo1, glyoxalase 1; Hagh, hydroxyacyl glutathione hydrolase; Hk, hexokinase; Ldh, lactate dehydrogenase; ns, not significant; Pdh, pyruvate dehydrogenase; PEP, Phosphoenolpyruvic acid; Pfk, phosphofructokinase; Pgk, phosphoglycerate kinase; Pgm1, phosphoglucomutase‐1; Pkm, pyruvate kinase isoenzyme; PKM2, pyruvate kinase isoenzyme 2; Tpi1, triosephosphate isomerase 1.

Renal phenotype of db/db mice was shown in Figure S1. Fasting blood glucose and body weight of db/db mice were significantly higher than that of db/m mice (Figure S1A,B). PAS staining demonstrated dilated mesangial matrix in the glomeruli of db/db mice (Figure S1C). Electron microscopy showed fused podocyte foot processes and thickened glomerular basement membrane in db/db mice (Figure S1D). ACR values of db/db mice were increased (Figure S1E). All these results indicated glomeruli and podocyte injury in 24‐week‐old db/db mice. By metabolomics, levels of DHAP, GAP, glucose‐6‐phosphate, and fructose 1, 6‐bisphosphate were significantly increased in glomeruli of db/db mice (Figure 1A), while their pyruvate and lactate levels were decreased (Figure 1A). Decrease of downstream glycolysis enzymes including LDHA and PKM2 protein expressions in glomeruli of db/db mice was observed by immunochemistry, immunofluorescence, and Western blotting (Figure 1B–F). Double immunofluorescence staining of LDHA/PKM2 and podocyte marker Synaptopodin revealed that both LDHA and PKM2 expressions in podocytes were downregulated as shown in Figure 1D,E. Similiarly, the protein expression pattern was confirmed by Western blotting (Figure 1F).

### 3.2 Oxidative stress, mTORC1 pathway activation and pyroptosis in glomeruli of db/db mice

**FIGURE 2:**
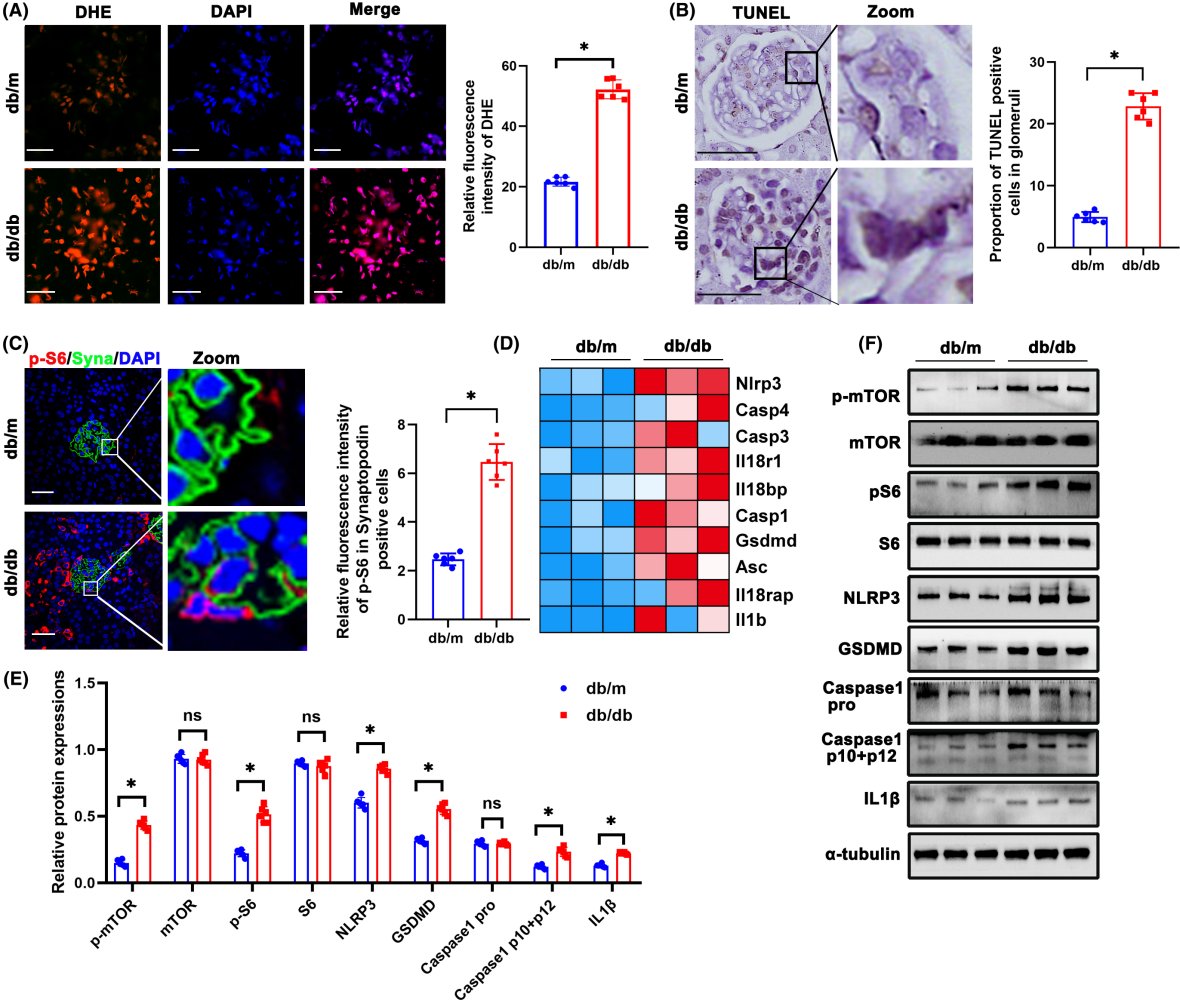
Oxidative stress, mTORC1 pathway activation and pyroptosis in glomeruli of db/db mice. (A) DHE staining to detect oxidative stress in glomeruli from different groups (original magnification, ×600). (B) Representative immunohistochemistry TUNEL staining of glomeruli in db/m and db/db mice. (C) Representative immunofluorescent staining of p‐S6 in podocyte of glomeruli from db/m and db/db mice. Synaptopodin was used as podocyte markers. (D) Gene expression profile was compared between glomeruli of db/m and db/db mice, and heat maps were generated based on expression of the genes related to pyroptosis. (E, F) Western blotting analysis of the expression of p‐mTOR, mTOR, p‐S6, S6, NLRP3, GSDMD, Caspase 1 pro, Caspase 1 p10 + p12 and IL‐1β in glomeruli from db/m and db/db mice. α‐tubulin was used as loading control. Scale bars: 20 μm. *n* = 6. *p < 0.05. DHE, dihydroethidium; GSDMD, gasdermin D; IL1β, interleukin 1 beta; mTOR, mechanistic target of rapamycin kinase; NLRP3, NLR family, pyrin domain containing 3; ns, not significant; p‐mTOR, phosphor mechanistic target of rapamycin kinase; p‐S6, S6 kinase; ROS, reactive oxygen species; S6, phosphor S6 kinase.

Oxidative Stress is a crucial pathogenic factor for podocyte damage in DKD.^7^ DHE staining showed that ROS production in glomeruli of db/db mice was significantly higher (Figure 2A). ROS is a fundamental factor in triggering pyroptosis by activating the NOD‐like receptor 3 (NLRP3) inflammasome.^20^ TUNEL staining showed higher proportion of TUNEL positive podocytes in db/db mice (Figure 2B). Double immunofluorescence staining of p‐S6 and Synaptopodin showed that p‐S6 expression was increased in podocytes (Figure 2C), which indicated activation of mTORC1 pathway. Transcriptome sequencing results of diabetic mice showed that expression of genes involved in pyroptosis including Nlrp3, Casp1, etc. was significantly higher in glomeruli of db/db mice (Figure 2D). Similarly, Western blotting showed increase of mTORC1 pathway and proteins of pyroptosis including NLRP3, GSDMD, Caspase1 and IL‐1β in the glomeruli of db/db mice (Figure 2E,F).

### 3.3 DHAP accumulation, ROS increase and pyroptosis activation in HG‐treated podocytes in vitro

**FIGURE 3:**
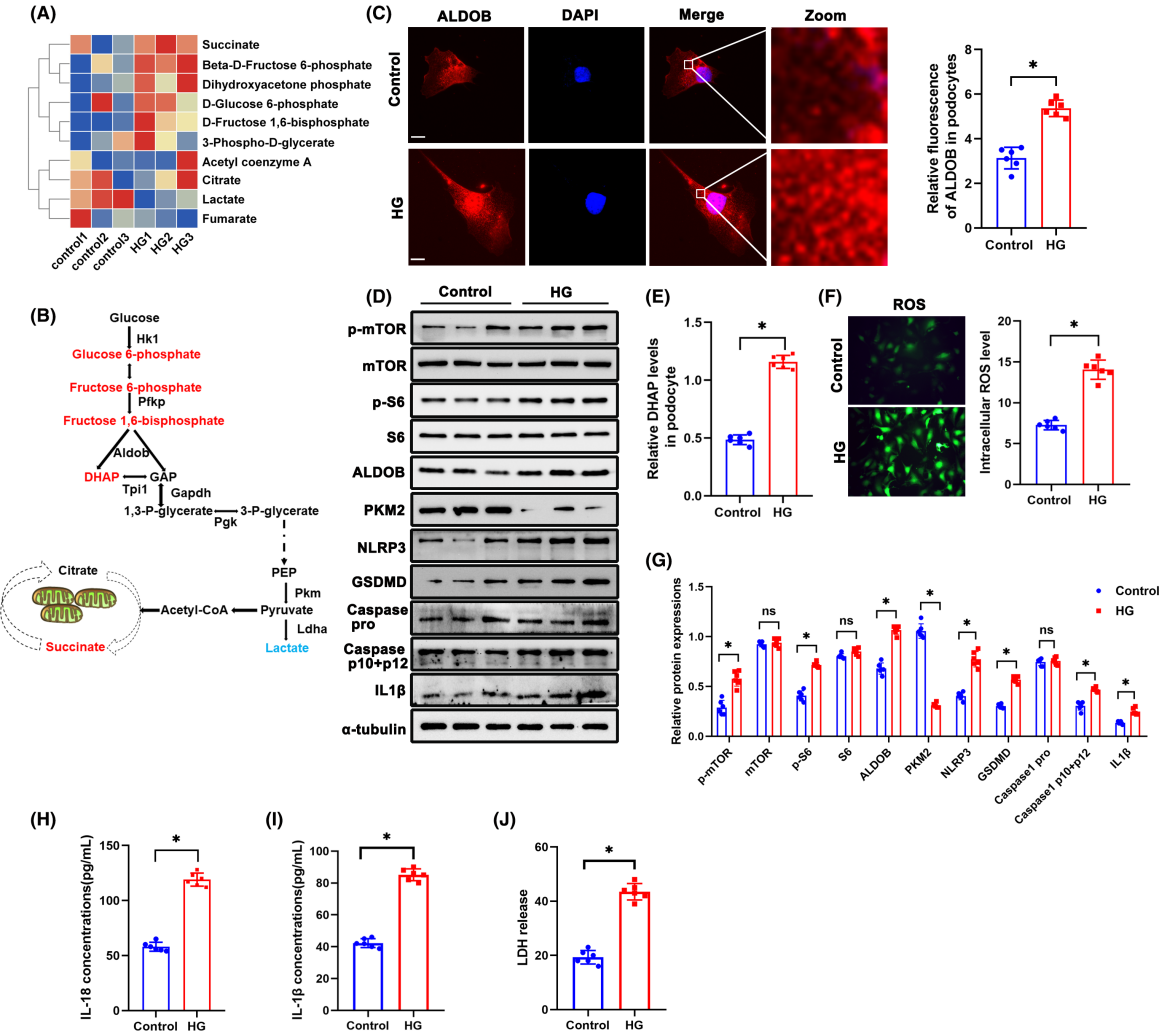
DHAP accumulation, ROS production increase and pyroptosis activation in HG‐treated podocytes in vitro. (A) Metabolomics of glomeruli of HG‐treated podocytes. Altered metabolites in glycolysis were showed in heat map. (B) A schematic flow illustrates the representative metabolites in glycolysis cascade. Letters in red represent upregulated metabolites and ones in blue represent downregulated metabolites from glomeruli of db/db mice in comparison to db/m mice. (C) Representative immunofluorescent staining of podocytes ALDOB in each group. (D) Western blotting analysis of the expression of p‐mTOR, mTOR, p‐S6, S6, ALDOB, PKM2, NLRP3, GSDMD, Caspase 1 pro, Caspase 1 p10 + p12 and IL1β in podocytes of each group. α‐tubulin was set as loading control. (E) DHAP levels in each group. (F) Representative DCFDA staining image of podocytes from each group (original magnification, ×40). (G) Quantification of protein expression. (H) IL‐18 levels in each group. (I) IL‐1β levels in each group. (J) LDH levels in each group. Scale bars: 10 μm. *n* = 6. *p < 0.05. ALDOB, aldolase B type; GSDMD, gasdermin D; HG, high glucose; IL1β, interleukin 1 beta; LDH, Lactate dehydrogenase; mTOR, mechanistic target of rapamycin kinase; NLRP3, NLR family, pyrin domain containing 3; ns, not significant; PKM2, pyruvate kinase isoenzyme 2; p‐mTOR, phosphor mechanistic target of rapamycin kinase; p‐S6, S6 kinase; ROS, reactive oxygen species; S6, phosphor S6 kinase.

Metabolomics of HG‐treated podocytes showed increased levels of DHAP, succinate, fructose 6‐phosphate, glucose 6‐ phosphate, and fructose 1, 6‐bisphosphate (Figure 3A,B). As the key enzyme that catalyses the production of DHAP, ALDOB expression was detected in cultured podocytes. Immunofluorescence staining demonstrated increased expression of ALDOB in HG‐treated podocytes (Figure 3C). Western blotting showed the increase of ALDOB as well (Figure 3D). DHAP levels in HG treated podocytes were further confirmed (Figure 3E). Moreover, ROS production in cultured podocytes was assessed by H2‐DCFDA, and after HG treatment, podocytes showed a significant increase in ROS (Figure 3F). Western blotting showed activation of mTORC1 pathway and increased pyroptosis in podocytes in vitro (Figure 3D,G). In addition, level of IL‐18, IL‐1β, and LDH were higher in HG group (Figure 3H–J).

### 3.4 Suppression and overexpression of ALDOB alleviated pyroptosis in HG‐treated podocytes

**FIGURE 4:**
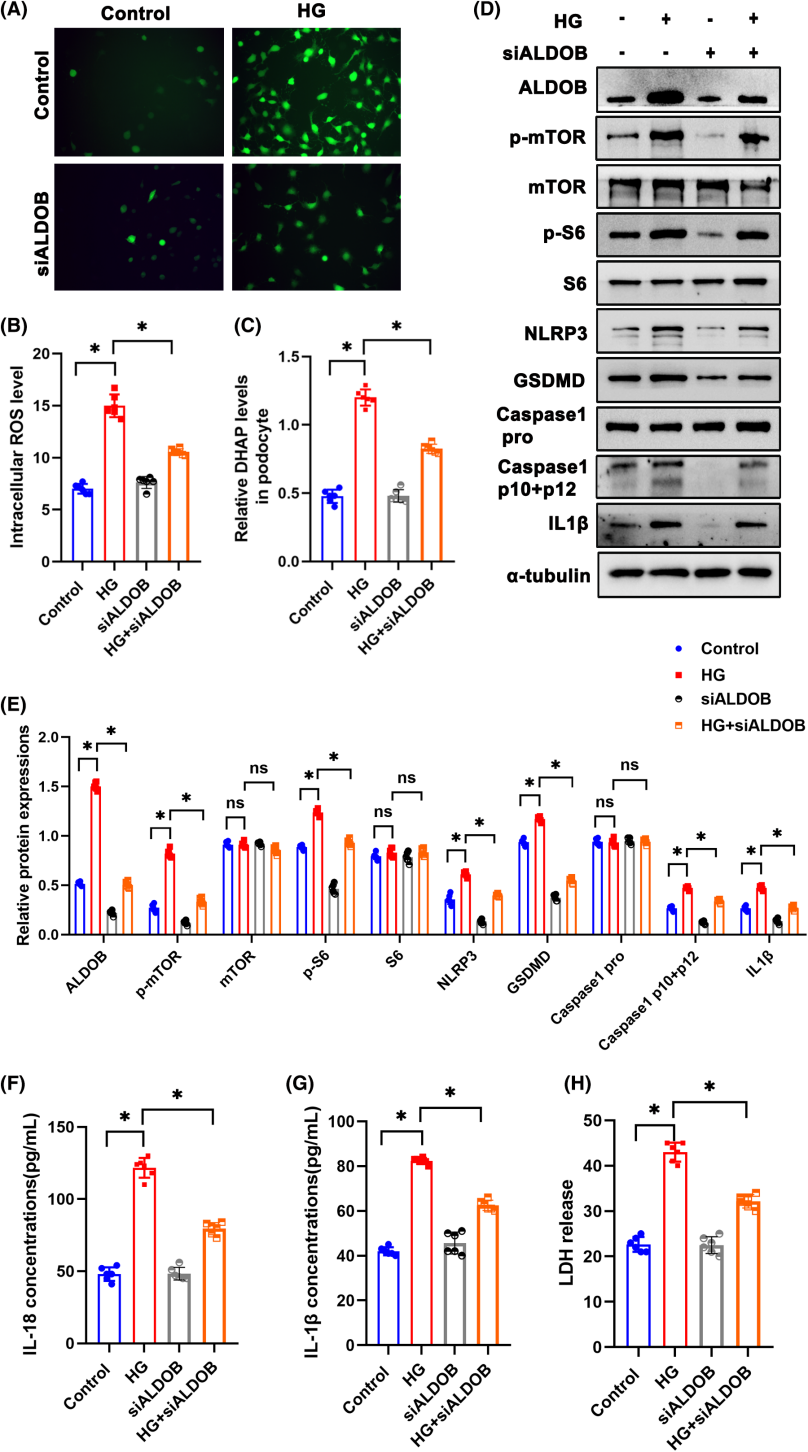
Suppression of ALDOB alleviated pyroptosis in HG‐treated podocytes. (A, B) Representative DCFDA staining image of podocytes from each group (original magnification, ×40). (C) DHAP levels in each group. (D, E) Western blotting analysis of the expression of p‐mTOR, mTOR, p‐S6, S6, ALDOB, NLRP3, GSDMD, Caspase 1 pro, Caspase 1 p10 + p12 and IL1β in podocytes of each group. α‐tubulin was set as loading control. (F) IL‐18 levels in each group. (G) IL‐1β levels in each group. (H) LDH levels in each group. Scale bars: 10 μm. *n* = 6. *p < 0.05. ALDOB, aldolase B type; GSDMD, gasdermin D; HG, high glucose; IL1β, interleukin 1 beta; LDH, Lactate dehydrogenase; mTOR, mechanistic target of rapamycin kinase; NLRP3, NLR family, pyrin domain containing 3; ns, not significant; p‐mTOR, phosphor mechanistic target of rapamycin kinase; p‐S6, S6 kinase; ROS, reactive oxygen species; S6, phosphor S6 kinase.

**FIGURE 5:**
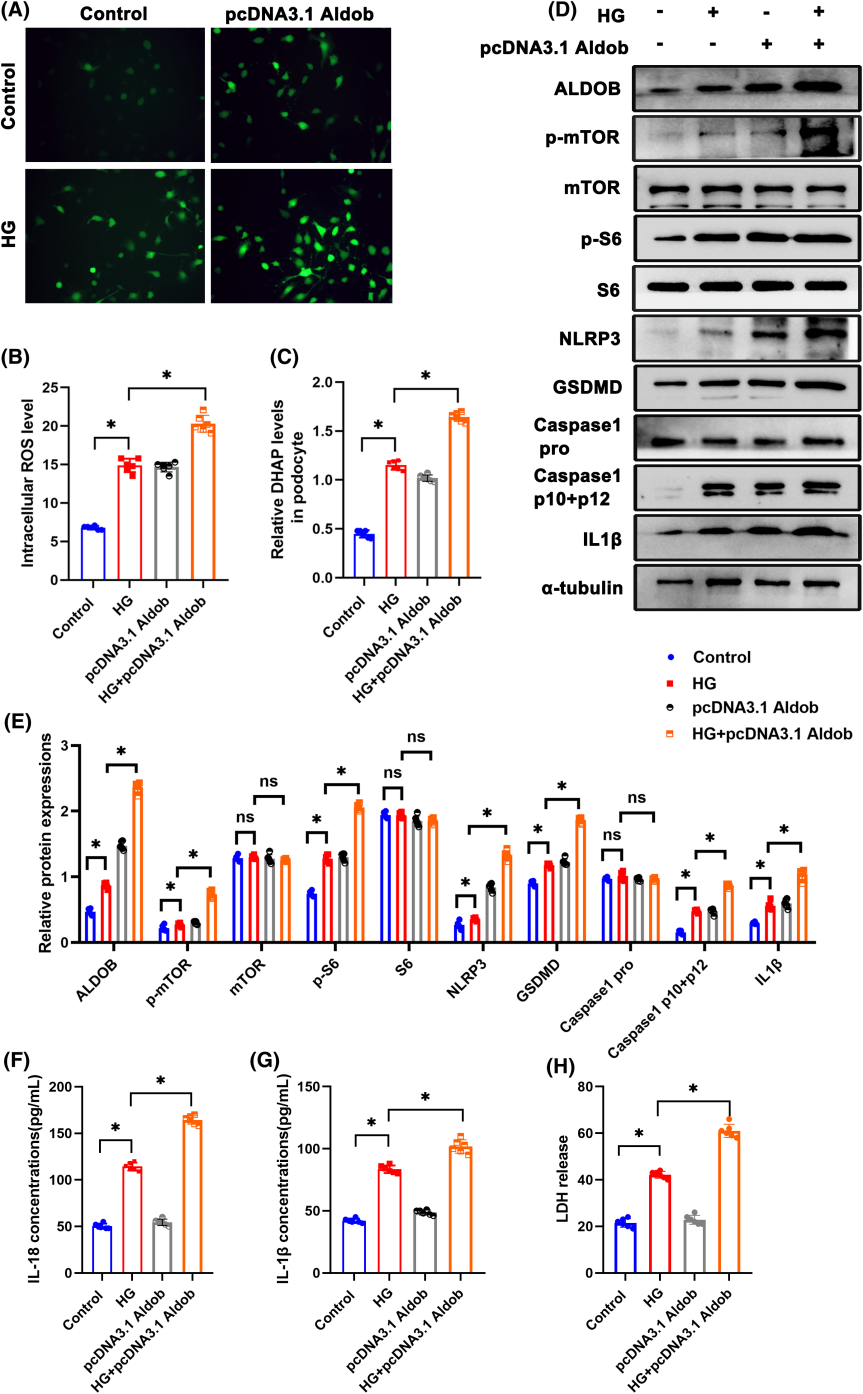
Overexpression of ALDOB accelerated pyroptosis in HG‐treated podocytes. (A, B) Representative DCFDA staining image of podocytes from each group (original magnification, ×40). (C) DHAP levels in each group. (D, E) Western blotting analysis of the expression of ALDOB, p‐mTOR, mTOR, p‐S6, S6, NLRP3, GSDMD, Caspase 1 pro, Caspase 1 p10 + p12 and IL1β in podocytes of each group. α‐tubulin was set as loading control. (F) IL‐18 levels in each group. (G) IL‐1β levels in each group. (H) LDH levels in each group. Scale bars: 10 μm. *n* = 6. *p < 0.05. ALDOB, aldolase B type; GSDMD, gasdermin D; HG, high glucose; IL1β, interleukin 1 beta; LDH, Lactate dehydrogenase; mTOR, mechanistic target of rapamycin kinase; NLRP3, NLR family, pyrin domain containing 3; ns, not significant; p‐mTOR, phosphor mechanistic target of rapamycin kinase; p‐S6, S6 kinase; ROS, reactive oxygen species; S6, phosphor S6 kinase.

To further investigate role of DHAP in podocytes, siALDOB was transfected. ROS production in podocytes was decreased in siALDOB transfection group (Figure 4A,B). DHAP levels in podocytes were significantly reduced in siALDOB group (Figure 4C). By Western blotting, p‐S6 and p‐mTOR protein levels were decreased in siALDOB group, indicating the inhibition of mTORC1 pathway (Figure 4D,E). Furthermore, siALDOB inhibited pyroptosis by suppressing NLRP3, GSDMD, Caspase1 and IL‐1β (Figure 4D,E). What's more, level of IL‐18, IL‐1β and LDH were significantly reduced siALDOB group than that of HG group (Figure 4F–H). Besides, pcDNA3.1 ALDOB was applied to overexpress ALDOB. Interestingly, ROS production in podocytes was increased by overexpression of ALDOB (Figure 5A,B). DHAP levels were lower in pcDNA3.1 ALDOB group (Figure 5C). Western blotting showed higher activation level of mTORC1 pathway and pyroptosis in podocytes (Figure 5D,E). Compared with HG group, level of IL‐18, IL‐1β and LDH were increased in pcDNA3.1 ALDOB group (Figure 5F–H).

### 3.5 GAP had no effect on mTORC1 pathway activity in podocytes

**FIGURE 6:**
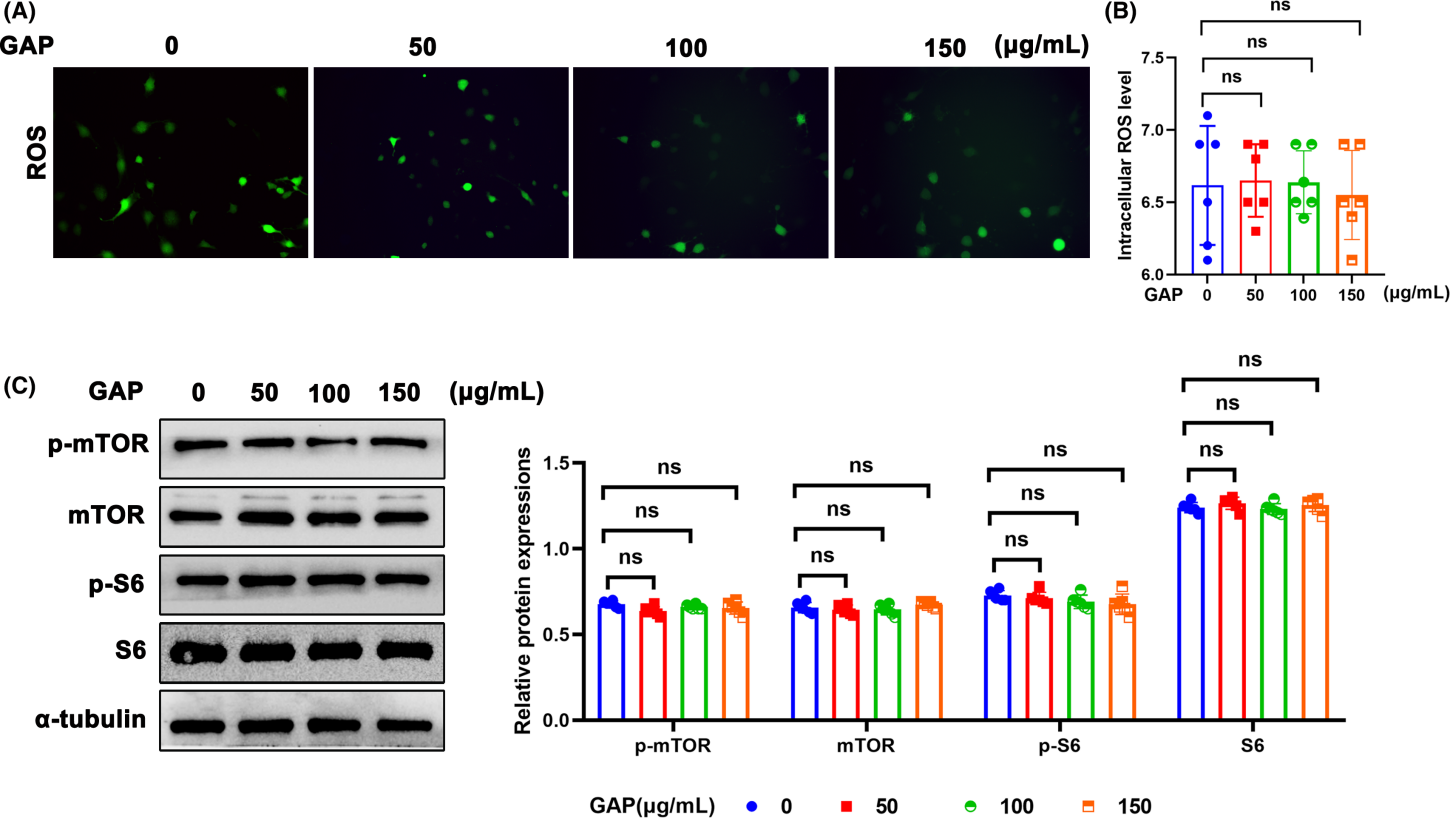
Effect of GAP on mTORC1 pathway activity in podocytes. (A, B) Representative DCFDA staining image of podocytes from each group (original magnification, ×40). (C) Western blotting analysis of the expression of ALDOB, p‐mTOR, mTOR, p‐S6, and S6 in podocytes of each group. α‐tubulin was set as loading control. *n* = 6. *p < 0.05. GAP, Glyceraldehyde 3 phosphate; mTOR, mechanistic target of rapamycin kinase; ns, not significant; p‐mTOR, phosphor mechanistic target of rapamycin kinase; p‐S6, S6 kinase; ROS, reactive oxygen species; S6, phosphor S6 kinase.

Aldolase cleaves fructose 1, 6‐bisphosphate into DHAP and GAP, and we found both DHAP and GAP levels in glomeruli under diabetic state were increased (Figure 1A). To investigate whether GAP was involved in the activation of mTORC1 pathway, GAP was added to stimulate podocytes. Surprisingly, different concentration of GAP from 0 to 150 μg/mL did not increase ROS production in podocytes (Figure 6A,B). Importantly, different concentration of GAP had no effect on mTORC1 pathway activity in podocytes as well by Western blotting (Figure 6C).

### 3.6 Rapamycin ameliorated pyroptosis in HG‐treated podocytes

**FIGURE 7:**
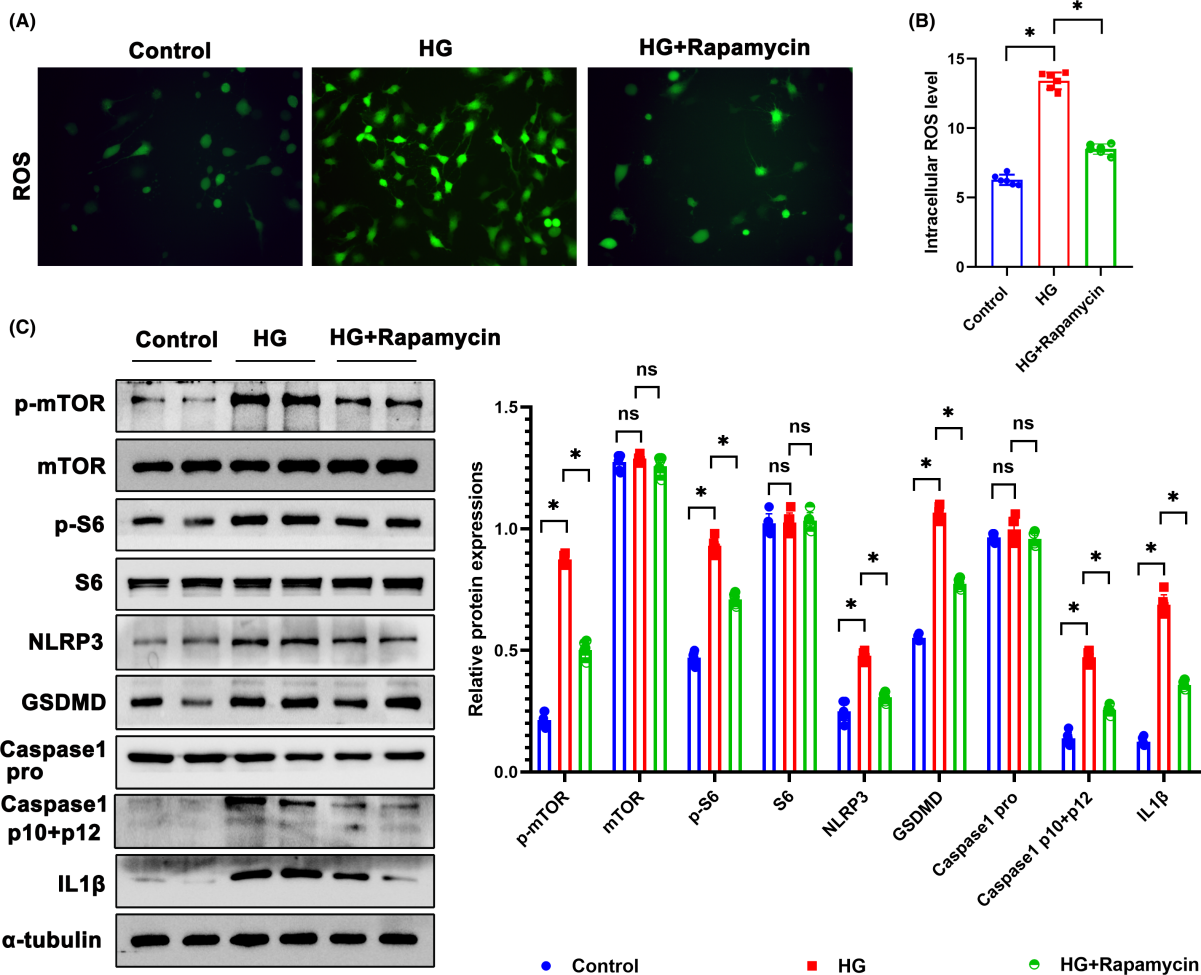
Rapamycin ameliorated pyroptosis in HG‐treated podocytes. (A, B) Representative DCFDA staining image of podocytes from each group (original magnification, ×40). (C) Western blotting analysis of the expression of p‐mTOR, mTOR, p‐S6, S6, NLRP3, GSDMD, Caspase 1 pro, Caspase 1 p10 + p12, and IL1β in podocytes of each group. α‐tubulin was set as loading control. Scale bars: 10 μm. *n* = 6, *p < 0.05. GSDMD, gasdermin D; HG, high glucose; IL1β, interleukin 1 beta; mTOR, mechanistic target of rapamycin kinase; NLRP3, NLR family, pyrin domain containing 3; ns, not significant; p‐mTOR, phosphor mechanistic target of rapamycin kinase; p‐S6, S6 kinase; ROS, reactive oxygen species; S6, phosphor S6 kinase.

Rapamycin is a widely used inhibitor of mTOR pathway. After rapamycin treatment, ROS production in podocytes was reduced (Figure 7A,B). Western blotting showed that rapamycin inhibited p‐S6 and p‐mTOR protein expression and ameliorated pyroptosis in HG‐treated podocytes (Figure 7C).

## 4 DISCUSSION

**FIGURE 8:**
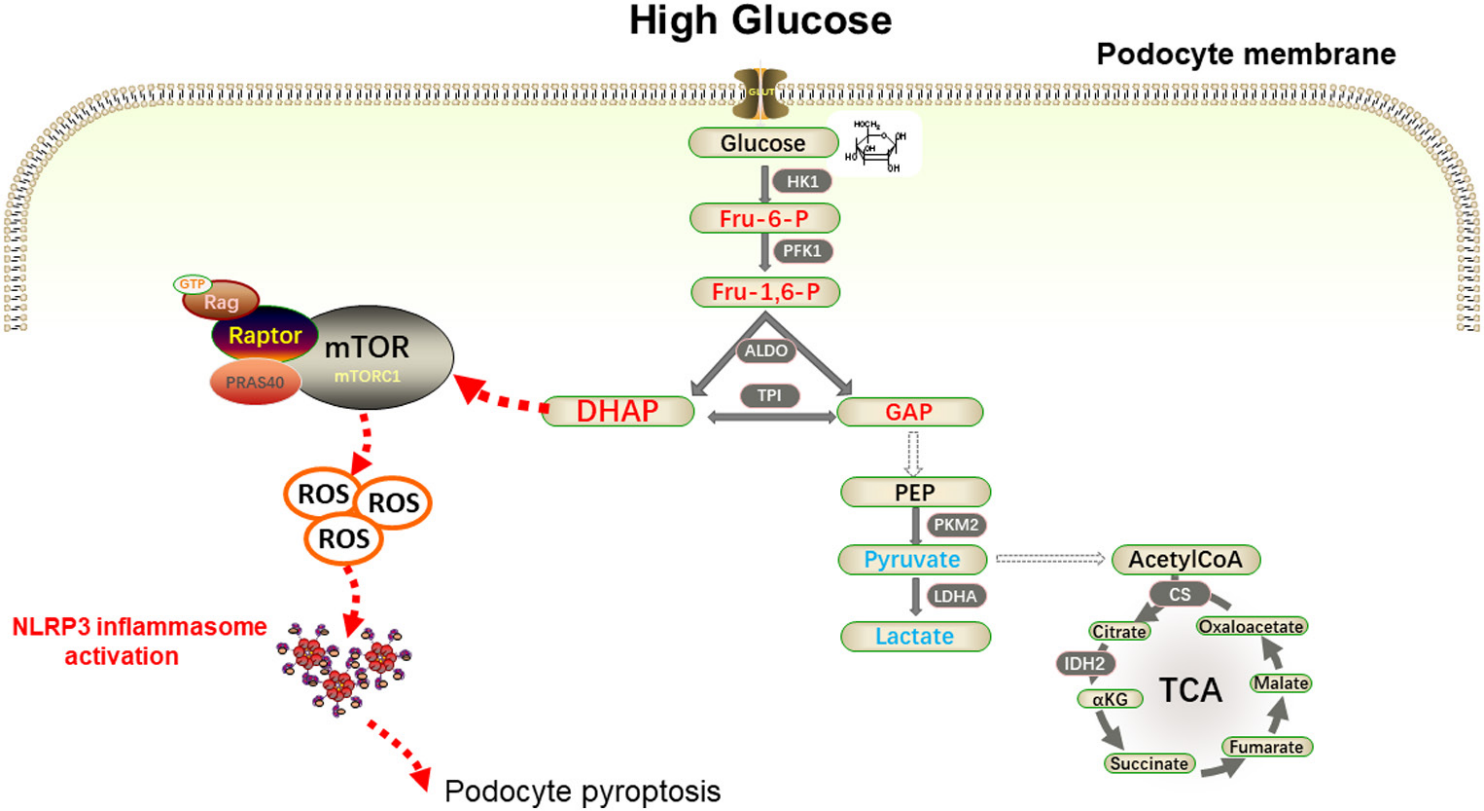
Accumulated glycolytic intermediate DHAP induces podocyte pyroptosis in DKD through activation of mTORC1/ROS/NLRP3 pathway.

This study revealed that DHAP, an intermediate product of glycolysis, activated mTORC1/ROS/NLRP3 pathway and induced podocyte pyroptosis in DKD. To the best of our knowledge, this is the first study to demonstrate the detrimental role of DHAP in podocytes in DKD (Figure 8).

A growing body of evidence suggests that mTORC1 activation is a critical factor in the development of DKD, which can cause damage to both podocytes and tubular cells.^11^ Despite this, the exact mechanism underlying mTORC1 activation in DKD remains unclear. Recent research has suggested that elevated levels of DHAP, a key metabolic metabolite, can signal to mTORC1 activation through the GATOR‐Rag signalling pathway.^18^ In DKD, hyperglycaemia is known to lead to an increase in DHAP levels in podocytes. This increase in DHAP can then result in mTORC1 activation, ultimately contributing to the damage caused by DKD. Further studies are needed to fully understand the relationship between mTORC1 activation and the development of DKD, as well as to identify potential therapeutic targets to mitigate the effects of this disease.

Studies suggest that the activation of mTORC1 in podocytes, which is associated with mitochondrial autophagy and biogenesis, can lead to the production of ROS.^21^, ^22^ Additionally, it has been shown that mTORC1, a metabolic signalling complex, can promote pyroptosis by controlling ROS production in mitochondria.^9^ In the present study, we found that mTORC1 activation in podocytes could induce ROS production and pyroptosis.

With the expansion of studies on DKD, much attention has been paid on the potential role of pyroptosis in DKD pathogenesis.^23^ A recent study showed that high glucose intervention promoted caspase‐11 and caspase‐4 expression and the decomposition of GSDMD. Moreover, the knockout of caspase‐11 or GSDMD could significantly improve the deterioration of renal function and injury of glomeruli and podocytes.^13^ The NLRP3 inflammasome was involved in the pathogenesis of many kidney diseases, including acute kidney injury and CKD.^24^ NLRP3 inflammasome could also promote both disease occurrence and progression in DKD. Additionally, ROS could initiate the activation of NLRP3 inflammasomes in diabetic state,^25^ and inhibition of NLRP3 inflammasome activation could inhibit renal inflammation and fibrosis via suppression of oxidative stress in DKD.^26^, ^27^ Clinical studies found that diabetic patients with proteinuria had significantly higher expression levels of IL‐1β, IL‐18 and NLRP3.^24^ Hence, it is crucial to find the key factors that leads to NLRP3 inflammasome activation and pyroptosis in podocytes in DKD. Here we demonstrated that DHAP/mTORC1/ROS played an essential role in podocyte pyroptosis in DKD, and inhibition of both DHAP production and mTORC1 activation reduced podocyte pyroptosis.

Metabolic disorder caused by hyperglycaemia has been a hit in the pathogenesis of DKD. It was reported that glycolysis played an important role in podocyte energy supply.^28^ Activation of pyruvate kinase M2 (PKM2), a key glycolytic enzyme, may protect against the progression of diabetic glomerular pathology.^29^ Yuan's study found that Pkm2‐knockdown podocytes showed reduction of energy metabolism, resulting in defects of cell differentiation. Podocyte‐specific Pkm2‐knockout (KO) mice developed worse albuminuria and podocyte injury after adriamycin treatment.^30^ Our recent study reported that overexpression of phosphofructokinase platelet type (PFKP) reduced podocyte injury in hyperglycaemia state.^19^ Study of Li, et al. suggested that hyperglycaemia could induce Smad4 localization to mitochondria in podocytes, resulting in reduced glycolysis and oxidative phosphorylation and increased production of ROS.^31^ These studies highlight the complex interplay between metabolic pathways and podocyte function in DKD and provide potential targets for the treatment of this condition.

However, glycolysis is an important metabolic pathology in podocyte from the perspective of both energy and function of the intermediate products. Our recent study also found that fructose 1, 6‐bisphosphate protected podocytes from cytoskeletal rearrangement in DKD.^19^ Otherwise, metabolites including sorbitol and fructose in polyol pathway contributed to podocyte foot process fusion and interstitial fibrosis of db/db mice.^32^ In the present study, we identified DHAP as an important metabolite that induced podocyte pyroptosis in DKD. Moreover, DHAP could be further converted to GAP by triosephosphate isomerase (TPI). In humans, TPI deficiency is a rare autosomal disorder, which causes haemolytic anaemia, neurological disease and even death due to block of the glycolysis pathway and accumulation of DHAP in red blood cells.^33^ Studies have also found that DHAP plays an essential role in the glycosylation process,^34^ and aberrant glycosylation has been connected with pathogenic signalling in podocytes.^35^ In addition, elevated DHAP levels can produce oxidative stress and impair mitochondrial dysfunction in human cells.^36^, ^37^

## 5 CONCLUSION

In conclusion, our study demonstrates for the first time that elevation of glucose metabolite DHAP exacerbated podocyte pyroptosis through mTORC1/ROS/NLRP3 pathway. Inhibition of glycolysis enzyme ALDOB and mTORC1 activation can protect podocyte from pyroptosis in diabetic condition. Our study highlights the importance of DHAP in podocyte pyroptosis and its potential as a therapeutic target for DKD. The identification of DHAP as a key inducer of podocyte pyroptosis underscores the importance of understanding the mechanisms underlying this process and developing effective interventions to mitigate its effects.

## AUTHOR CONTRIBUTIONS

**Zongwei Zhang:** Data curation (equal); formal analysis (equal); investigation (equal); methodology (equal); resources (equal). **Hongtu Hu:** Data curation (equal); investigation (equal); methodology (equal); project administration (equal); resources (equal). **Qiang Luo:** Investigation (equal); methodology (equal). **Keju Yang:** Resources (equal); software (equal). **Zhengping Zou:** Funding acquisition (equal); methodology (equal); supervision (equal). **Ming Shi:** Supervision (equal); writing – review and editing (equal). **Wei Liang:** Funding acquisition (equal); project administration (equal); supervision (equal); writing – review and editing (equal).

## CONFLICT OF INTEREST STATEMENT

The authors confirm that there are no conflicts of interest.

## Supporting information


Data S1.

Data S2.

Figure S1.

## Data Availability

The data that support the findings of this study are available from the corresponding author upon reasonable request.
