# Supplementary material for: Dihydroxyacetone phosphate accumulation leads to podocyte pyroptosis in diabetic kidney disease
Source: J Cell Mol Med. 2023 Dec 8;28(3):e18073. doi: 10.1111/jcmm.18073 (PMC10844688; doi:10.1111/jcmm.18073)
Supplement: Supplementary file 3 — Figure S1. [file JCMM-28-e18073-s001.docx]

Supplementary Figure1

Figure1


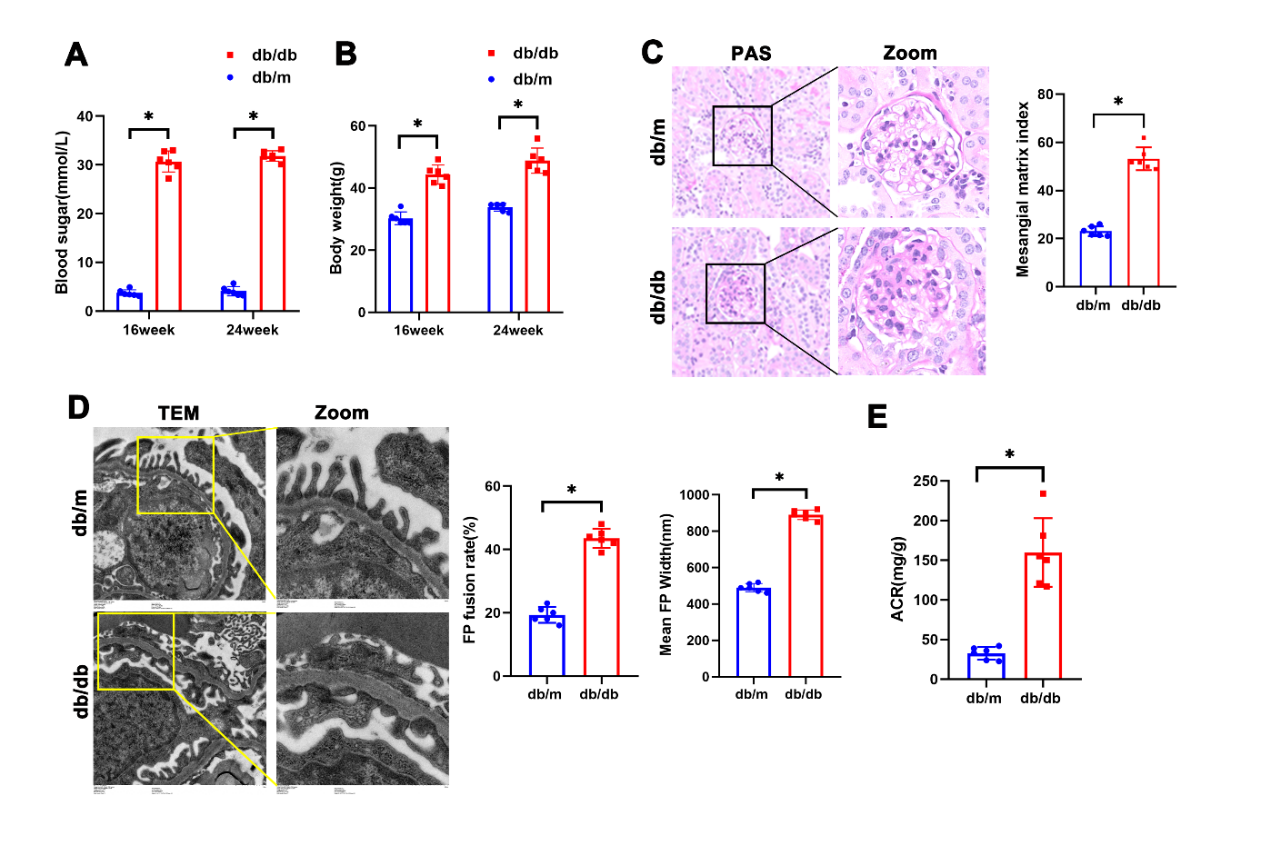


**Figure 1. Renal phenotype of db/db mice**

(A) Blood sugar levels of db/m and db/db mice. (B) Body weight of db/m and db/db mice. (C) Representative microscopy images and quantification of PAS staining of kidney sections for db/m and db/db mice at 24 weeks of age (original magnification ×400). (D) Representative findings of the ultrastructure of capillary loops by transmission electron microscopy from db/m and db/db mice at different timepoint (original magnification×8,000, ×12,000). (E) Quantitative analysis of ACR (albumin-to-creatinine ratio) in db/m and db/db mice. n=6. **P*< 0.05, ns: not significant. Scale bars: 20 µm.
